# Lysozyme-coated silver nanoparticles for differentiating bacterial strains on the basis of antibacterial activity

**DOI:** 10.1186/1556-276X-9-565

**Published:** 2014-10-11

**Authors:** Sumaira Ashraf, Mariyam Asghar Chatha, Wardah Ejaz, Hussnain Ahmed Janjua, Irshad Hussain

**Affiliations:** 1 Department of Chemistry, SBA School of Science & Engineering (SSE), Lahore University of Management Sciences (LUMS), DHA, Lahore Cantt 54792, Pakistan; 2 Current address: Fachbereich Physik Philipps Universität Marburg Renthof 7, D-35037 Marburg, Germany; 3 Department of Environmental Sciences, Kinnaird College for Women, Lahore 54800, Pakistan; 4 Department of Biology, SBA School of Science & Engineering (SSE), Lahore University of Management Sciences (LUMS), DHA, Lahore Cantt 54792, Pakistan; 5 Current address: Atta-ur-Rehman School of Applied Biosciences, National University of Science and Technology (NUST), Islamabad 44000, Pakistan

**Keywords:** Lysozyme, Silver nanoparticles, Antimicrobial activity, Colony-forming units, Bacterial strains differentiation

## Abstract

Lysozyme, an antibacterial enzyme, was used as a stabilizing ligand for the synthesis of fairly uniform silver nanoparticles adopting various strategies. The synthesized particles were characterized using UV-visible spectroscopy, FTIR, dynamic light scattering (DLS), and TEM to observe their morphology and surface chemistry. The silver nanoparticles were evaluated for their antimicrobial activity against several bacterial species and various bacterial strains within the same species. The cationic silver nanoparticles were found to be more effective against *Pseudomonas aeruginosa* 3 compared to other bacterial species/strains investigated. Some of the bacterial strains of the same species showed variable antibacterial activity. The difference in antimicrobial activity of these particles has led to the conclusion that antimicrobial products formed from silver nanoparticles may not be equally effective against all the bacteria. This difference in the antibacterial activity of silver nanoparticles for different bacterial strains from the same species may be due to the genome islands that are acquired through horizontal gene transfer (HGT). These genome islands are expected to possess some genes that may encode enzymes to resist the antimicrobial activity of silver nanoparticles. These silver nanoparticles may thus also be used to differentiate some bacterial strains within the same species due to variable silver resistance of these variants, which may not possible by simple biochemical tests.

## Background

Nanomaterials composed of metal nanoparticles are attracting significant attention because of their enormous applications in photonics [1], electronics [2], optoelectronics [3], catalysis [4,5], sensing [6], antimicrobial products [7,8], pharmaceuticals [9], and therapeutics [10,11]. Currently, more than 50% of the inventoried antimicrobial consumer products comprise nanosilver due to their cost effectiveness and broad-spectrum antibacterial and antifungal properties. These nanosilver-based antimicrobial products include fabrics, bandages, and food containers as deodorizers and disinfectants [12]. Serious efforts are being made to explore the use of silver nanoparticles for the disinfection of drinking water [13] and as pesticide to control pests attack on various crops [14]. The antibacterial activity of silver nanoparticles is usually considered because of leached silver ions that affect the microbial growth either by inhibiting DNA replication [15,16] or by inactivating cellular proteins [17]. Silver nanoparticles also interact with the bacterial cell membrane causing structural changes and damage which ultimately result in the cell death [8].

Several protocols have been reported for the synthesis of silver nanoparticles, such as borohydride reduction [18], acrylate/citrate reduction [19-22], microwave irradiation [23-26], photoreduction [27], polyol process [28,29], plant extracts and vegetable oils [30-35], vitamins [36], amino acids [24], polymers and proteins [37-39]. These silver nanoparticles exhibit varying degrees of antimicrobial activity depending on their size, shape, and surface chemistry. Coating silver nanoparticles with cationic ligands and other antimicrobial molecules can further enhance their antimicrobial activity due to the synergistic effect [37]. For example, lysozymes that damage bacterial cell walls by catalyzing the hydrolysis of 1,4-beta-linkages between *N*-acetyl-d-glucosamine and *N*-acetylmuramic acid residues present in peptidoglycan layer surrounding the bacterial cell membrane [40] have recently been used as catalyst for the synthesis of silver nanoparticles/nanoclusters using different strategies [41]. However, the formation of metal nanoparticles in pure protein system is a bit challenging and requires exquisite crystal growth control depending on the sequence, size, conformation, and charge of the protein in the system. Recently, a few other proteins such as casein [38] and bovine serum albumin have also been reported to produce gold/silver nanoparticles/clusters [42-46] for different applications.

In the present study, we have produced lysozyme-coated silver nanoparticles by various strategies and examined their antibacterial activity against six different bacterial species. For evaluating the efficacy of these nanoparticles against different strains of the same species, we have also examined the antibacterial activities of these nanoparticles against different strains from the same species. In bacteria, many antibiotic-resistant genes are present on plasmids, transposons, or integrons that can act as vectors to transfer these genes through horizontal gene transfer (HGT) to other strains of the same bacterial species, as well as to bacteria in another genus or species; thus, many bacterial strains from the same species develop resistance against antibiotics [47,48]. We have selected various bacterial species in order to examine the antibacterial activity of lysozyme-stabilized silver nanoparticles (Ag NPs). Various bacterial strains from the same species were further tested for their response to Ag NPs, and the results were compared based on the MIC (minimum inhibitory concentration) of silver nanoparticles. Interestingly, heat-denatured, lysozyme-stabilized silver nanoparticles were found to be more bactericidal compared to those coated with the native enzyme. Moreover, in case of some strains from the same species, not only different bactericidal potential of Ag NPs was observed but also some strains were totally resistant against these Ag NPs, at least under the given experimental conditions. We have verified our results by counting the colony-forming units (CFU) of bacteria in the presence of different concentrations of native and heat-denatured, enzyme-caped Ag NPs and positive (lysozyme solution) and negative (microbial strain in Luria-Bertani (LB) media without Ag NPs and lysozyme) controls. The difference in antimicrobial activity of these particles is due to the variation in pan genome (pan genome contains core genome and the dispensable genome; the dispensable genome is present in some strains of the same bacterial species [49]) which has led to the conclusion that the silver nanoparticle-based antimicrobial products may not be equally effective against all the bacterial strains of the same species. These particles may further be used in differentiating some strains within the same bacterial species due to variable silver resistance of these variants which may not be possible by simple biochemical tests.

## Methods

### Materials

Silver nitrate, lysozyme (from chicken egg white, approximately 100,000 units/mg), sodium chloride, and sodium hydroxide were purchased from Sigma-Aldrich (St. Louis, MO, USA). Sodium borohydride was purchased from VWR International Limited (BDH Merck Limited, Poole Dorset, UK). Tryptone, yeast extract, and agar were purchased from MP Biomedicals (Solon, OH, USA). The pure microbial strains were collected from local hospital and were identified by biochemical tests. LB was used as growth media for cultivating microbial strains and consisted of 0.5% yeast extract and 1% tryptone and sodium chloride in water having a pH of 7. Ultrapure water with a resistivity of 18.2 MΩ cm was used as solvent in all preparations.

### Methodology

#### Synthesis of different types of lysozyme-coated Ag NPs

**Synthesis of lysozyme-coated Ag NPs in alkaline medium (AL Ag NPs)** In a typical reaction, 50 mL of 1 mM AgNO_3_ solution in various flasks was placed in an incubator shaker set at 37°C at 180 rpm. A 5 mL of lysozyme solution (1 mg/mL) prepared by dissolving the enzyme in different molar concentrations of sodium hydroxide solutions (ideally 0.6 M) was added into each flask and incubated under the above-mentioned reaction conditions. The formation of yellowish/brownish suspension of silver nanoparticles was observed after 1 day. The UV-visible absorption spectra of the particles formed were recorded regularly on daily basis until 17 days.

**Synthesis of lysozyme-Ag NPs by borohydride reduction method (BL Ag NPs)** A 0.75 mL of lysozyme solution (1 mg/mL H_2_O) was added into 10 mL of 1 mM AgNO_3_ solution under vigorous stirring and continued stirring for 1 h. After 1 h, 0.25 mL of freshly prepared NaBH_4_ solution (1 mg/mL H_2_O) was added under vigorous stirring. The reaction contents were stirred vigorously for at least 4 h to ensure the completion of the reaction. In order to examine the effect of the concentration of lysozyme and NaBH_4_ on nanoparticles formation, 0.5, 0.75, 1, 1.25, 1.5, and 2 mL of lysozyme solution (1 mg/mL H_2_O) and 0.05, 0.1, 0.25, 0.5, 0.75, and 1 mL of NaBH_4_ solution (1 mg/mL H_2_O) were used while keeping the silver content constant.

**Synthesis of lysozyme-Ag NPs by reflux method (RL Ag NPs)** Two milligrams of lysozyme was dissolved in 20 mL of 1 mM AgNO_3_ aqueous solution under vigorous stirring and subjected to reflux at approximately 120°C. After 10 to 15 min of reflux, 1 mL of 1 M NaOH solution was added, and the reflux was continued for another hour under continuous stirring. Different amounts of lysozyme (1 to 3 mg) and sodium hydroxide (0.5 to 2 mL) were used while optimizing the reaction conditions, and 2 mg of lysozyme and 1 mL of 1 M NaOH per 20 mL of 1 mM AgNO_3_ solution were found to be optimum.

**Microwave-assisted synthesis of lysozyme-Ag NPs (ML Ag NPs)** A 0.5 mL of lysozyme solution (1 mg/mL; 0.5 to 2 M NaOH) was added to 5 mL of 1 mM AgNO_3_ solution while stirring and irradiated with microwaves for 10 min in a microwave synthesizer (CEM Corporation, Matthews, NC, USA) at 50°C to 90°C under 250 psi. Yellowish brown solution of silver nanoparticles was obtained after the completion of reaction under optimum conditions (lysozyme 1 mg/ml; NaOH 1.5 M, 0.5 mL and T =90°C).

The silver nanoparticles formed by all four protocols described above were purified to remove the excess enzyme and other soluble impurities using ultracentrifugal filters (Amicon® Ultracentrifugal filters by Millipore Corporation (Billerica, MA, USA) having molecular weight cut-off value of 30 kDa operating at 2,000 rpm for 15 min). The purified and concentrated Ag NPs were then lyophilized and stored as dry powder for further analysis and used in subsequent experiments. Aqueous solution of lysozyme was subjected to similar reaction conditions as described above in various protocols except the addition of silver nitrate and stored as dry powder after lyophilization to be used as positive control in subsequent experiments.

#### Antibacterial activity of lysozyme-stabilized Ag NPs

Six bacterial species (*Pseudomonas aeruginosa*, *Staphylococcus aureus*, *Bacillus subtilis*, *Salmonella enterica*, *Escherichia coli*, and *Klebsiella pneumoniae*) were selected to examine their sensitivity to silver nanoparticles. For some species, more than one strain (four *P. aeruginosa*, two *S. enterica*, two *E. coli*, and two *K. pneumoniae*) were selected to check their nanoparticles' sensitivity. These bacterial strains were collected from Jinnah Hospital, Lahore, Pakistan from wound, pus, or fecal samples of patients suffering from bacterial infections and identified using various biochemical tests such as gram staining and strip tests (API 20E Biomerieux Inc., Durham, NC, USA). The bacterial culture (20 μL) was collected from exponentially grown culture and spread on LB agar plates. A 20 μL of all types of Ag NP suspensions was spotted on bacterial lawn from concentrations ranging from 1,000, 100, 10, 1, 0.1, and 0.01 ppm. The concentrations of lysozyme used as reactant corresponding to the above-mentioned concentrations of Ag NPs, subjected to similar reaction conditions, were used as positive control. All plates were incubated at 37°C for 24 h, and zones of inhibition (diameter of clear/bacteria free zones in mm) were measured. Maximum bactericidal activity was observed against *Pseudomonas aeruginosa* 3. This strain was selected for further studies, and the zones of inhibition of all above-mentioned Ag NPs were measured by the same method as described above. The concentrations of Ag NPs used were 100, 80, 60, 40, 20, and 10 ppm, and their corresponding lysozyme concentrations used as reactants were applied as positive control. CFU of this strain were counted in the presence of different doses of Ag NPs (100, 80, 60, 40, 20, 10, and 1 ppm) and their corresponding positive (lysozyme solutions) and negative (bacterial culture in the absence of any substance) controls. For CFU counting, bacterial strains were grown at 37°C for 24 h in LB media at 150 rpm, in the presence of all types of lysozyme-stabilized Ag NPs with their respective positive and negative controls. After 24 h, all cultures (containing all types of Ag NPs and their respective controls) were diluted (serial dilutions), and 1 mL of the selected dilutions was spread on LB agar plates. The plates were then incubated at 37°C for 24 h. Number of visible colonies were counted and multiplied by dilution factors to obtain the actual number of bacterial colonies in the original growth media. Using CFU counting method, we compared the bactericidal activity of all lysozyme-stabilized Ag NPs and that of the positive controls and compared those with bacterial populations growing in the LB media. All experiments were performed in triplicate. In order to further confirm the bactericidal/bacteriostatic activity of these nanoparticles, movies of *Pseudomonas aeruginosa* 3 were recorded in the absence (negative control) and presence (80 ppm) of all types of Ag NPs and their positive controls after an hour of incubation using Olympus CX41 light microscope coupled with DXC-390P 3CCD color video camera (Olympus Corporation, Shinjuku, Tokyo, Japan).

### Characterization of nanoparticles

Transmission electron microscopy (TEM) analysis of lysozyme-Ag NPs was carried out using high-resolution TEM (JEOL, JEM-3010; JEOL Ltd., Tokyo, Japan) operating at 300 kV. Nanoparticle specimens for inspection by TEM were prepared by slow evaporation of a drop of a dilute aqueous solution of the particles on a carbon-coated copper mesh grid. ImageJ software was used to calculate particle size distribution from transmission electron micrographs. The particle size distribution was determined using Zetasizer Nano ZS (Malvern Instruments, Worcestershire, UK). The surface chemistry of Ag NPs was determined using Bruker Alpha-P Fourier transform infrared (FTIR) with a diamond ATR attachment (Bruker Corporation, Billerica, MA, USA). UV-visible spectra of Ag NPs suspension were recorded using an Agilent 8453 UV-visible spectrophotometer (Agilent Technologies, Sta. Clara, CA, USA). Olympus CX41 light microscope coupled with DXC-390P 3CCD color video camera 12 V-0.62A (Sony Corporation, Tokyo, Japan) was used for observing and recording the effect of different concentrations of lysozyme and lysozyme-Ag NPs on bacteria.

## Results and discussion

Silver is a known antimicrobial agent in the form of ions or reduced metal nanoparticles [50-54] that are usually incorporated in a variety of consumer products to render them antimicrobial. Due to the antimicrobial activity of silver, it is very effective against several microbial strains [55-58] by disrupting microbial membrane and cell lysis. In the current study, we have tested the effect of silver nanoparticles on several bacterial species and observed that their antimicrobial activity is variable for different strains even from the same species. This variable resistance for silver nanoparticles from strains of the same bacterial species can be correlated to silver-resistant genes that may have acquired through HGT.

**Figure 1:**
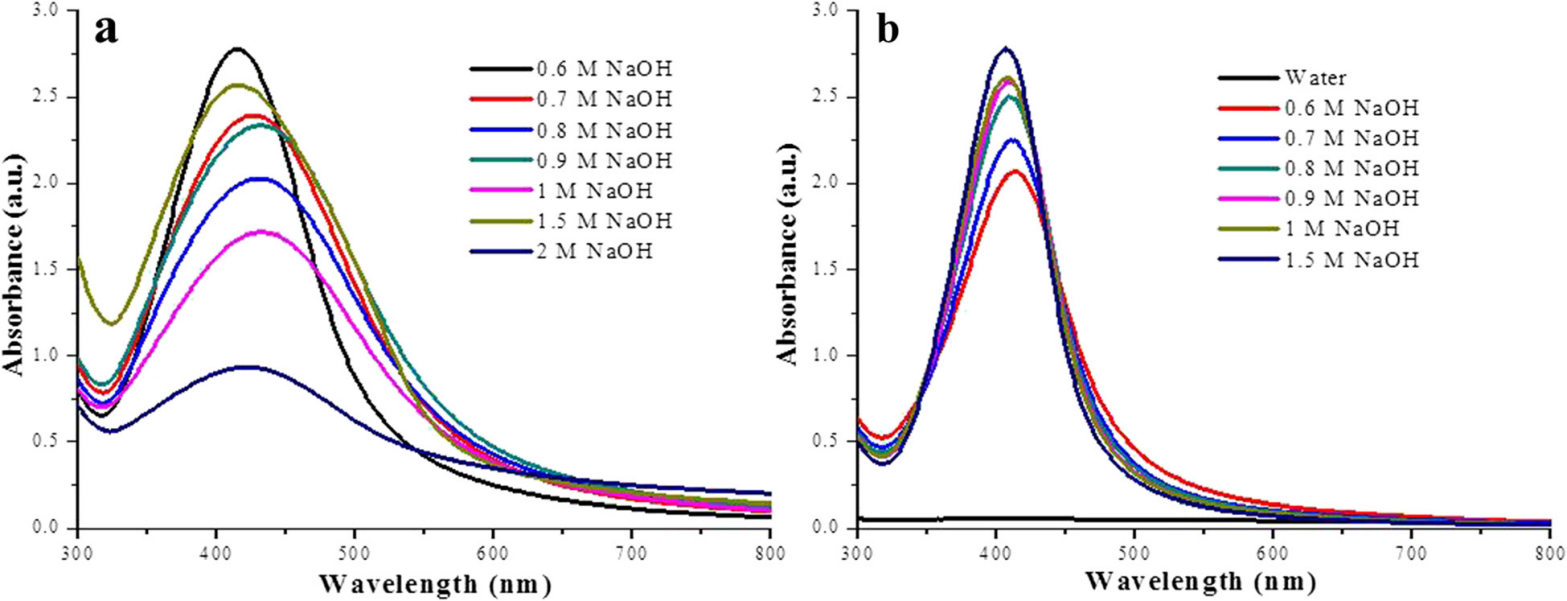
UV-visible absorption spectra of AL Ag NPs (a) and ML Ag NPs (b).

**Figure 2:**
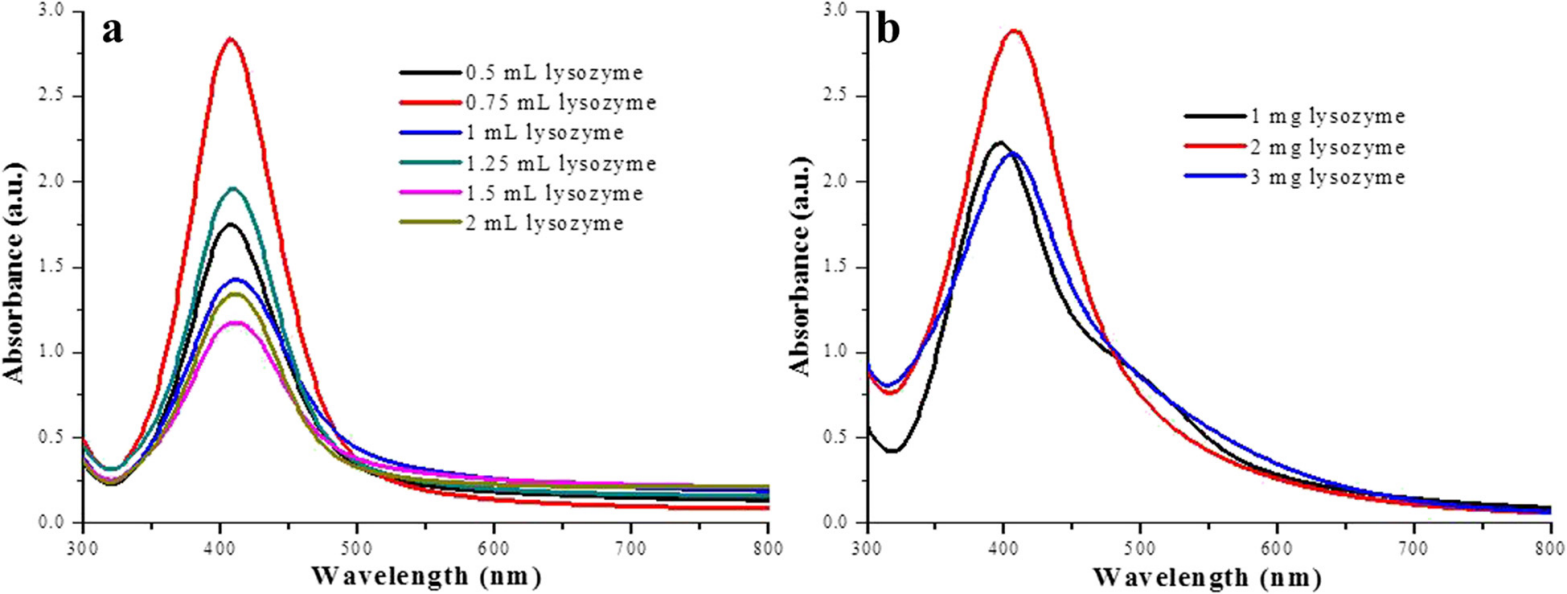
UV-visible absorption spectra of BL Ag NPs (a) and RL Ag NPs (b).

Silver nanoparticles coated with lysozyme had a peculiar yellowish brown color and were recognized by their characteristic surface plasmon resonance (SPR) band. By carefully monitoring the appearance of SPR band, the optimum reaction conditions were determined for the synthesis of lyso-Ag NPs. For AL Ag NPs, 0.6 M NaOH was found to be optimum among all the tested concentrations of base while keeping the concentration of the lysozyme constant. Their UV-visible absorption spectra were recorded on daily basis for several days and on day 17, SPR bands of maximum intensity were observed. Representative SPR bands of sliver nanoparticles are shown in Figure 1, where all the tested concentrations of base are compared for the synthesis of uniform Ag NPs using optimum concentration of base. For comparing the formation of AL Ag NPs using all tested concentrations of base in day-wise fashion, see Additional file 1: Figures S1 and S2. From the shape and intensity of SPR band for BL Ag NPs, 0.75 mL of lysozyme (Figure 2) and 0.25 mL of sodium borohydride (1 mg/mL H_2_O) (Additional file 1: Figure S3) per 10 mL of 1 mM AgNO_3_ were found to be optimum to form fairly uniform silver nanoparticles. In case of RL Ag NPs, 2 mg lysozyme (Figure 2) and 1 mL of 1 M sodium hydroxide (Additional file 1: Figure S4) per 20 mL of 1 mM AgNO_3_ were found ideal for the synthesis of NPs. Whereas, for ML Ag NPs, 1.5 M NaOH gave the best yield of fairly uniform Ag NPs while keeping the concentration of lysozyme and silver nitrate constant (Figure 1).

**Figure 3:**
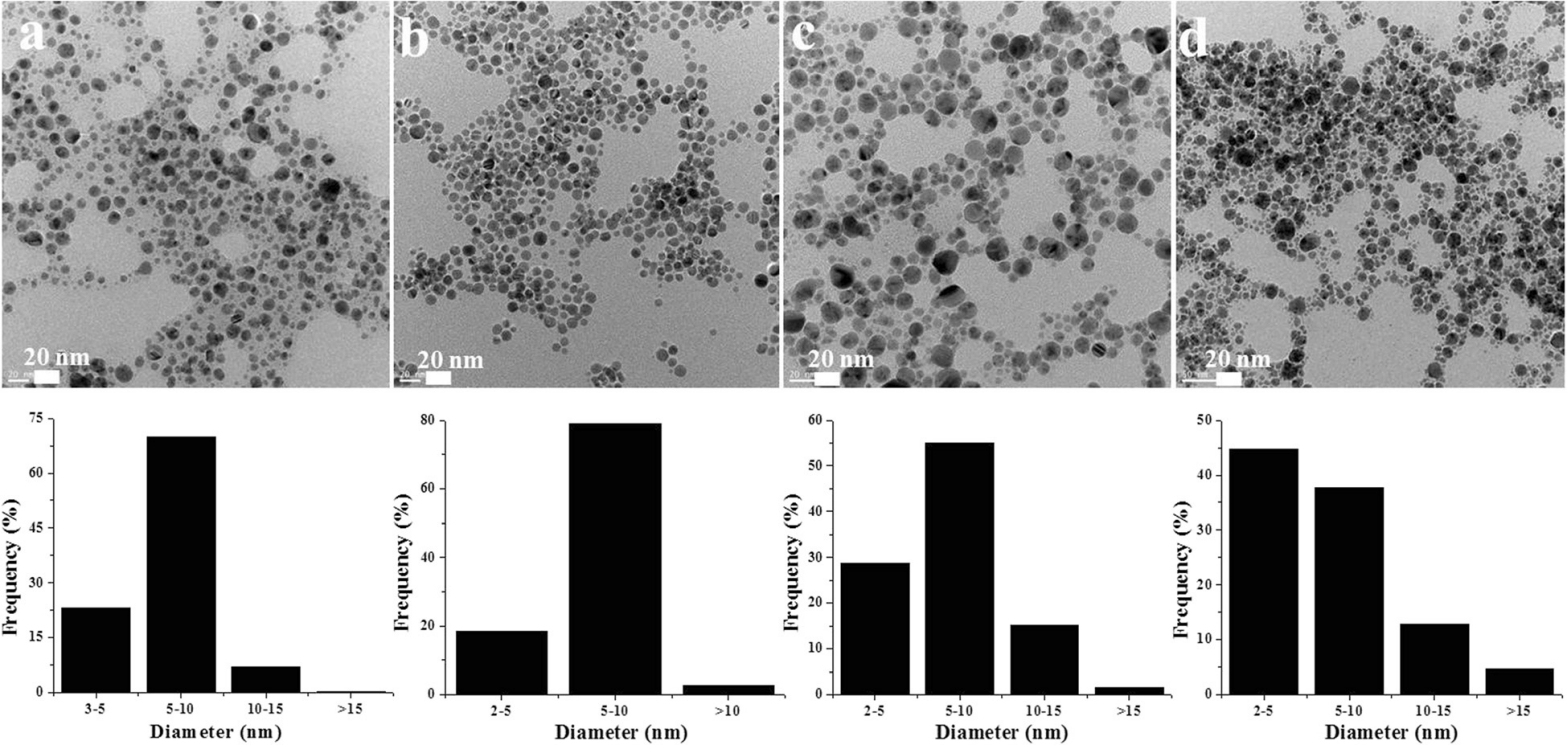
Transmission electron micrographs of AL Ag NPs (a), BL Ag NPs (b), RL Ag NPs (c), and ML Ag NPs (d) and their corresponding size distribution.

TEM is a useful characterization tool for determining the morphology of nanoparticles. For analysis of size distribution from transmission electron micrographs, at least 300 particles were analyzed using imageJ program to calculate the actual size and size distribution of Ag NPs formed by all above-mentioned recipes. Most of the particles were 2 to 15 nm in size and were spherical in shape (Figure 3). But most uniform and small-sized particles were formed by borohydride reduction method, i.e., BL Ag NPs. In order to calculate the size of particles along with the capping ligand, dynamic light scattering (DLS) measurements were also performed. A few representative transmission electron micrographs along with DLS size distribution for all types of Ag NPs are shown in Additional file 1: Figures S5 to S8. From DLS size distribution graphs, most of the particles were found to be in the range of 10 to 25 nm in diameter.

**Figure 4:**
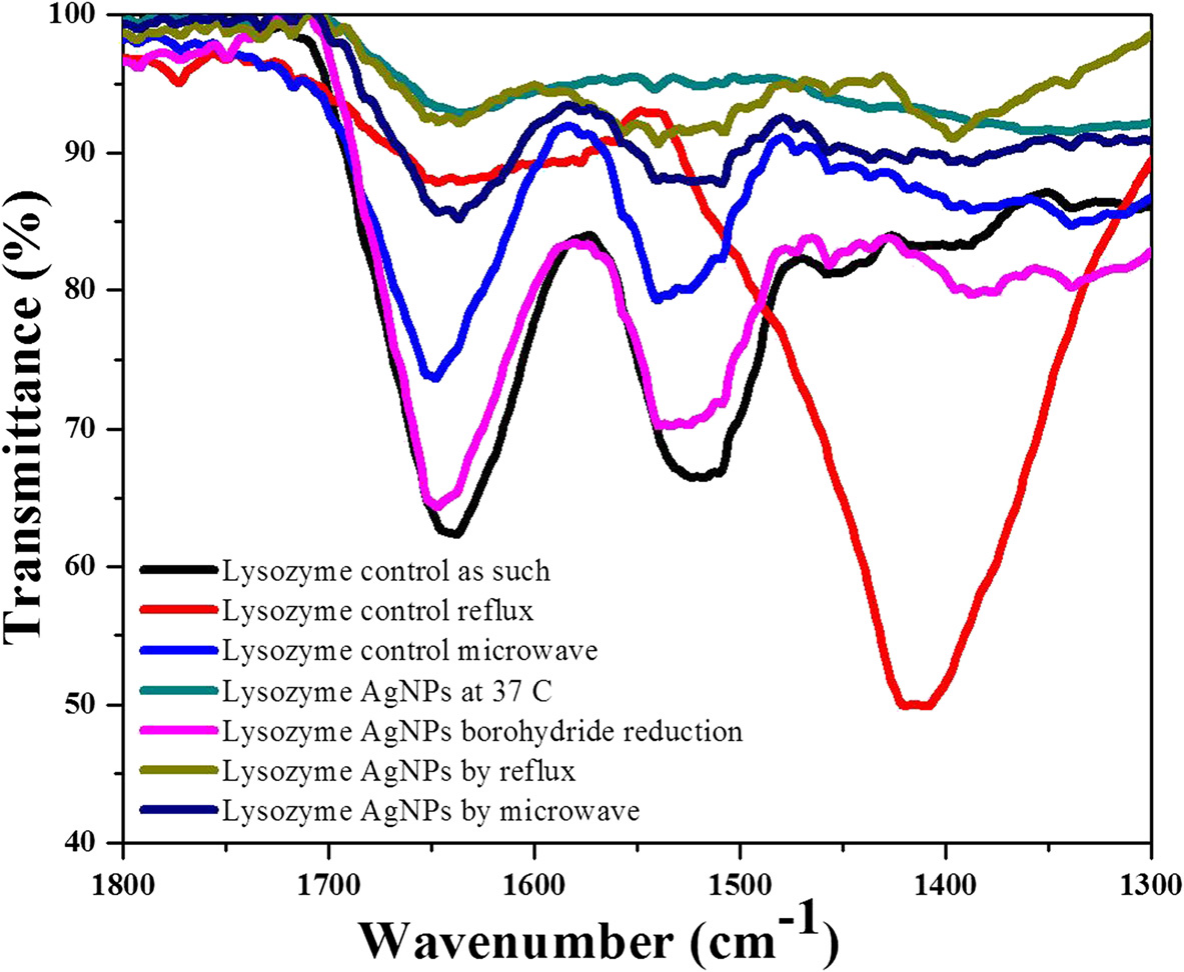
FTIR spectra of lysozyme-stabilized Ag NPs in the presence of native and heat-denatured lysozyme.

In order to determine the surface chemistry of Ag NPs, FTIR is usually considered as a useful spectroanalytical tool. FTIR spectroscopy is also a valuable tool to monitor the secondary structural nature of proteins in various environments [59] and is thus helpful to characterize proteins-coated metal nanoparticles. The positions of the amide I (C = O stretch, 1,700 to 1,600 cm^-1^) and amide II (C-N stretch and N-H deformation, 1,560 to 1,530 cm^-1^) and their band frequencies can be easily correlated to the structure of proteins. The specific stretching and bending vibrations of the peptide backbone in amide I, II, and III bands provide useful information about different types of secondary structures such as α-helix, β-sheets, turns, and unordered structures. Of all the amide bands of the peptide group, amide I has proven to be the most sensitive probe of protein secondary structure. The FTIR spectra of native and heat-denatured lysozymes and that of the particles formed from them (Figure 4) reveal that heat denaturation by means of reflux for 1 h totally shifted the positions of amide I band from 1,650 and 1,530 cm^-1^ towards 1,400 cm^-1^, indicating the disappearance of alpha-helical contents and the appearance of more β-laminar zones and intra-molecular β-sheets. While in the case of microwave treatment (90°C for 10 min), the intensity of amide peaks at 1,680 and 1,580 cm^-1^ became less intense, showing an increase in β-sheets secondary structures and a subsequent decrease in alpha-helical content at elevated temperature. These results are in agreement with the previous studies of determining the structure of native and heat-denatured lysozyme by means of FTIR analysis [60]. In the case of nanoparticles formation by native and heat-denatured lysozyme, the amide I peak is either decreased in intensity (disappeared) or shifted from its original position showing the interaction of lone pair of electrons on nitrogen of amide I in stabilization and formation of Ag NPs. Moreover, denaturation by means of thermal treatment exposes more cysteine residues by breaking disulfide bridges present in the molecules of lysozyme [61]. The exposed -SH groups of cysteine residues show high affinity towards silver hence helped in the interaction, stabilization, and formation of stable silver nanoparticles in higher yield in case of reflux and microwave treatments.

**Figure 5:**
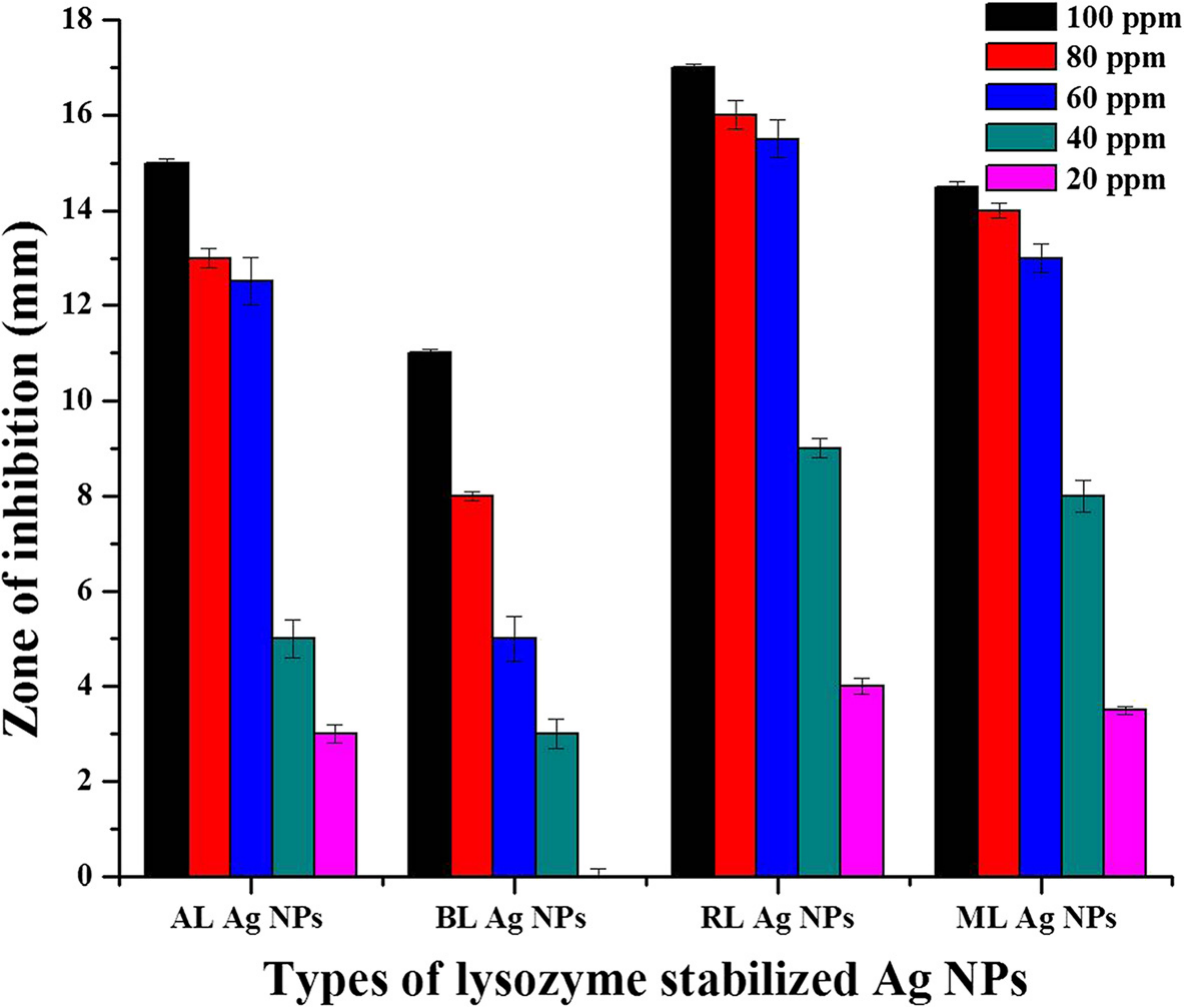
**Bactericidal effect of lysozyme-stabilized Ag NPs against *P. aeruginosa *3.** Zones of inhibition were measured in millimeters, whereas concentration of particles applied are given in parts per million. (No zone formation was observed at any dose of positive control (lysozyme solution). However, clear zone formation was observed in the presence of lysozyme-stabilized Ag NPs at 10-ppm concentration.

In order to explore and compare the antimicrobial potential of lyso-Ag NPs, we studied their bactericidal effect against several bacterial strains of the same species. Some strains were resistant against silver and were not affected even at 1,000 ppm concentration of Ag NPs (*E. coli 2*), while some showed intermediate resistance e.g., *K. pneumoniae* 2, *S. enterica* 2 and 1 (Table 1). *P. aeruginosa* 3 was the most sensitive strain as indicated by the formation of maximum zones of inhibition (mm) in the presence of all four types of lyso-Ag NPs applied. The difference in antimicrobial action of Ag NPs against different strains of the same species may be due to the presence of genes that are resistant to silver nanoparticles that may have evolved through HGT that ultimately leads to genome plasticity in bacterial strains of the same species [47]. In microorganisms, many of the accessory genes are acquired through horizontal transfer. These accessory genes are recognized as genomic islands (GEIs) which are discrete DNA segments between closely related strains of the same species. GEIs have major impact on genome plasticity that ultimately leads to diversification and adaptation of microorganisms. This genome plasticity between bacterial strains of the same species may result in antibiotic resistance, acquisition of virulence genes, and formation of new catabolic pathways [48]. The most sensitive strain, i.e., *P. aeruginosa* 3, was selected to examine the effect of low concentrations (100 to 10 ppm) of lyso-Ag NPs (Figure 5). At 10 ppm concentration of Ag NPs, no clear zone of inhibition was observed with any of the four types of lyso-Ag NPs used. The mechanism of antimicrobial activity of Ag NPs varies depending upon the size, shape, and surface chemistry of Ag NPs and the genomics and physiology of the microbial strain under investigation (gram positive/negative and genome variability, etc.). However, it is known that silver increases the permeability of bacterial membranes resulting in the collapse of membrane potential thus affecting ion transport processes and the depletion of intercellular ATP [17,62]. In order to further understand the mechanism of antimicrobial activity and to produce silver nanoparticles with enhanced antimicrobial activity, we have investigated the integration of antimicrobial activity of lysozyme from hen egg white with that of silver nanoparticles. Heat denaturation by means of reflux enhanced the antimicrobial potency of lyso-Ag NPs, followed by denatured ML Ag NPs and native AL Ag NPs. Poor antimicrobial activity was observed in case of BL Ag NPs; zones of inhibitions (mm) are shown in Figure 5. The enhanced bactericidal effect of Ag NPs coated with heat-denatured enzyme was further confirmed by counting their CFU given in Additional file 1: Table S1. The CFU of positive control were almost identical to the negative control, showing inability of the lysozyme to suppress bacterial growth at low concentration. In case of RL Ag NPs, no CFU were observed even at 40 ppm concentration. At Ag NP concentrations below 20 ppm, the bacteria started to grow as in negative control experiments containing only the growth medium without Ag NPs. From these results, we have concluded that the antimicrobial activity of these Ag NPs is in the order RL Ag NPs > ML Ag NPs > AL Ag NPs > BL Ag NPs (Figure 5, Table 1; Additional file 1: Table S1). The enhanced bactericidal effect of heat-denatured, lysozyme-stabilized Ag NPs is in agreement with the findings of Ibrahim et al. [61]. The bactericidal effect of heat-denatured lysozyme against gram-negative bacteria is enhanced due to more exposed hydrophobic residues (tryptophan residues) of enzyme, leading to the more disruption of gram-negative bacterial cell walls. The FTIR spectra (Figure 4) of heat-denatured lysozyme show more content of β-sheets/structures compared to those in native enzyme. This conformation may thus enhance the antimicrobial activity of heat-treated lyso-Ag NPs by exposing more hydrophobic groups (hydrophobic tryptophan residues) to interact with the walls of gram-negative bacteria. Videos of lysozyme-treated *P. aeruginosa* 3 and those treated with all types of Ag NPs (80 ppm) after 1 h of incubation are given in Additional file 1: Videos S1 to S9. There is a clear difference in the motility of bacteria, in the case of negative and positive controls (they were clearly motile) as compared to Ag NPs-treated bacteria (they tended to be sessile). From these results, it can be inferred that enhanced antimicrobial properties are conferred to the antimicrobial products containing silver nanoparticles but there may be many bacterial species, and even bacterial strains within the same species, that show partial or complete resistance against silver nanoparticles due to the genomic variation associated with HGT. This implies that all silver nanoparticles-based antimicrobial products may not be equally effective in suppressing the bacterial growth. Moreover, lyso-Ag NPs may also be used to differentiate some strains of the same bacterial species that are difficult to be differentiated by the biochemical tests, based upon the difference of their antimicrobial activity.

**Table 1:** Bactericidal effect of lysozyme-stabilized Ag NPs against different bacterial strains

**Bacterial strains**	**Type of lysozyme-stabilized Ag NPs**							
	**AL Ag NPs**		**BL Ag NPs**		**RL Ag NPs**		**ML Ag NPs**	
	**1,000 ppm**	**100 ppm**	**1,000 ppm**	**100 ppm**	**1,000 ppm**	**100 ppm**	**1,000 ppm**	**100 ppm**
*P. aeruginosa* 1	14.7 ± 0.09	12.7 ± 0.09	11 ± 0.08	9.25 ± 0.1	13.5 ± 0.15	11.35 ± 0.12	14 ± 0.07	11.5 ± 0.09
*P. aeruginosa* 2	14.8 ± 0.1	12.7 ± 0.15	11.67 ± 0.11	11 ± 0.09	15.5 ± 0.13	11.67 ± 0.11	15 ± 0.14	11.5 ± 0.12
*P. aeruginosa* 3	18 ± 0.15	15 ± 0.14	14 ± 0.17	11.67 ± 0.20	19 ± 0.16	14.5 ± 0.13	17 ± 0.15	13.5 ± 0.125
*P. aeruginosa* 4	12 ± 0.04	5 ± 0.04	10 ± 0.04	0 ± 0.04	11 ± 0.04	0 ± 0.04	10 ± 0.04	0 ± 0.04
*S. aureus*	14.5 ± 0.3	11.67 ± 0.35	11.17 ± 0.24	5 ± 0.27	16 ± 0.33	12 ± 0.28	14 ± 0.34	12.83 ± 0.31
*B. subtilis*	14 ± 0.22	10.67 ± 0.26	10.33 ± 0.29	9.5 ± 0.25	15 ± 0.23	12 ± 0.21	13.5 ± 0.28	13 ± 0.27
*S. enterica* 1	12.5 ± 0.19	0 ± 0.18	10.5 ± 0.16	0 ± 0.20	16.5 ± 0.17	12 ± 0.22	15.5 ± 0.15	13 ± 0.15
*S. enterica* 2	13.5 ± 0.15	0 ± 0.14	11 ± 0.19	0 ± 0.15	16 ± 0.16	0 ± 0.13	14 ± 0.21	0 ± 0.18
*E. coli* 1	13 ± 0.23	10.33 ± 0.25	11.17 ± 0.19	10 ± 0.18	15 ± 0.21	11 ± 0.24	14.67 ± 0.18	11.5 ± 0.19
*E. coli* 2	0 ± 0.17	0 ± 0.24	0 ± 0.19	0 ± 0.22	0 ± 0.23	0 ± 0.22	0 ± 0.021	0 ± 0.25
*K. pneumoniae* 1	12.17 ± 0.15	10 ± 0.19	10 ± 0.19	8.83 ± 0.16	14.33 ± 0.17	11.16 ± 0.18	16 ± 0.15	12 ± 0.19
*K. pneumoniae* 2	9 ± 0.21	5 ± 0.19	5 ± 0.18	0 ± 0.20	15 ± 0.17	0 ± 0.19	16.5 ± 0.22	0 ± 0.23

Zone diameter in millimeter, concentrations of particles applied in parts per million.

## Conclusions

Lysozyme-stabilized silver nanoparticles were synthesized by developing various strategies in aqueous media. These nanoparticles were characterized by means of UV-visible and FTIR spectroscopy, DLS and transmission electron microscopy. The particles showed different antimicrobial action against various bacterial species and various bacterial strains of the same species. Some bacterial strains from the same species (*E. coli* 1) were found sensitive to these particles whereas others were highly resistant to silver nanoparticles (*E. coli* 2). Among all the species tested, one gram-negative strain (*P. aeruginosa 3*) was highly affected by lyso-Ag NPs. Heat denaturation enhanced the bactericidal activity of lysozyme-coated Ag NPs as compared to native enzyme-coated Ag NPs. By means of biochemical tests, identification of bacterial species is possible leaving strains within the species unidentified. Lyso-Ag NPs differentiated some of the strains within the same bacterial species based upon the difference of their antimicrobial activity.

## Competing interests

The authors declare that they have no competing interests.

## Authors' contributions

SA and IH conceived and designed all the experiments. SA and MA performed the experiments under the guidance of IH and HJ. All authors discussed the interpretation of results. SA and IH co-wrote the draft paper and HJ helped in the revision of the manuscript. Correspondence and requests for materials should be addressed to IH. All authors read and approved the final manuscript.

## Supplementary Material

Additional file 1 **Supplementary materials.** Lysozyme- coated silver nanoparticles for differentiating bacterial strains on the basis of antibacterial activity. Click here for file
