# Supplementary material for: Lysozyme-coated silver nanoparticles for differentiating bacterial strains on the basis of antibacterial activity
Source: Nanoscale Res Lett. 2014 Oct 11;9(1):565. doi: 10.1186/1556-276X-9-565 (PMC4242785; doi:10.1186/1556-276X-9-565)
Supplement: Additional file 1 — Supplementary materials. Lysozyme- coated silver nanoparticles for differentiating bacterial strains on the basis of antibacterial activity. [file 1556-276X-9-565-S1.zip › 1260298150140483_add1.docx]

Lysozyme Stabilized Silver Nanoparticles for Differentiating Bacterial Strains on the Basis of Antibacterial Activity

*Sumaira Ashraf,^a , b^Mariyam Asghar,^c^ Wardah Ejaz,^a^ Hussnain A Janjua,^d^ and Irshad Hussain^a*^*

^a^ Department of Chemistry, SBA School of Science & Engineering (SSE), Lahore University of Management Sciences (LUMS), DHA, Lahore Cantt – 54792, Pakistan. ^b^ (current address) Fachbereich Physik and WZMW Philipps Universität Marburg Renthof 7, D-35037, Marburg, Germany. ^c^ Department of Environmental Sciences, Kinnaird College for Women, Lahore, Pakistan. ^d^ Atta-ur-Rehman School of Applied Biosciences, National University of Science & Technology (NUST), Islamabad, Pakistan.

* Corresponding author: Professor Irshad Hussain

Email: [ihussain@lums.e du.pk](mailto:ihussain@lums.e%20du.pk); [irshadnibge@gmail.com](mailto:irshadnibge@gmail.com)

Tel: +92 42 3560 8133

Fax: +92 42 3560 8314

**Abstract**

Lysozyme, an antibacterial enzyme, was used as a stabilizing ligand for the synthesis of fairly uniform silver nanoparticles adopting various strategies. The synthesized particles were characterized using UV-visible spectroscopy, FTIR, dynamic light scattering (DLS), and TEM to observe their morphology and surface chemistry. The silver nanoparticles were evaluated for their antimicrobial activity against several bacterial species and strains within the same species. The cationic silver nanoparticles were found to be more effective against *Pseudomonas aeruginosa 3* compared to other bacterial species/ strains investigated. Some of the bacterial strains of the same species showed variable antibacterial activity. The difference in antimicrobial activity of these particles has led to the conclusion that antimicrobial products formed from silver nanoparticles may not be equally effective against all the microbes. This difference in the antimicrobial activity of silver nanoparticles for different strains from the same species may be due to the genome islands that are acquired through Horizontal Gene Transfer (HGT). These genome islands are expected to possess some genes that may encode enzymes to resist antimicrobial activity of silver nanoparticles.

**Keywords**: Lysozyme, silver nanoparticles, antimicrobial activity, colony forming units.


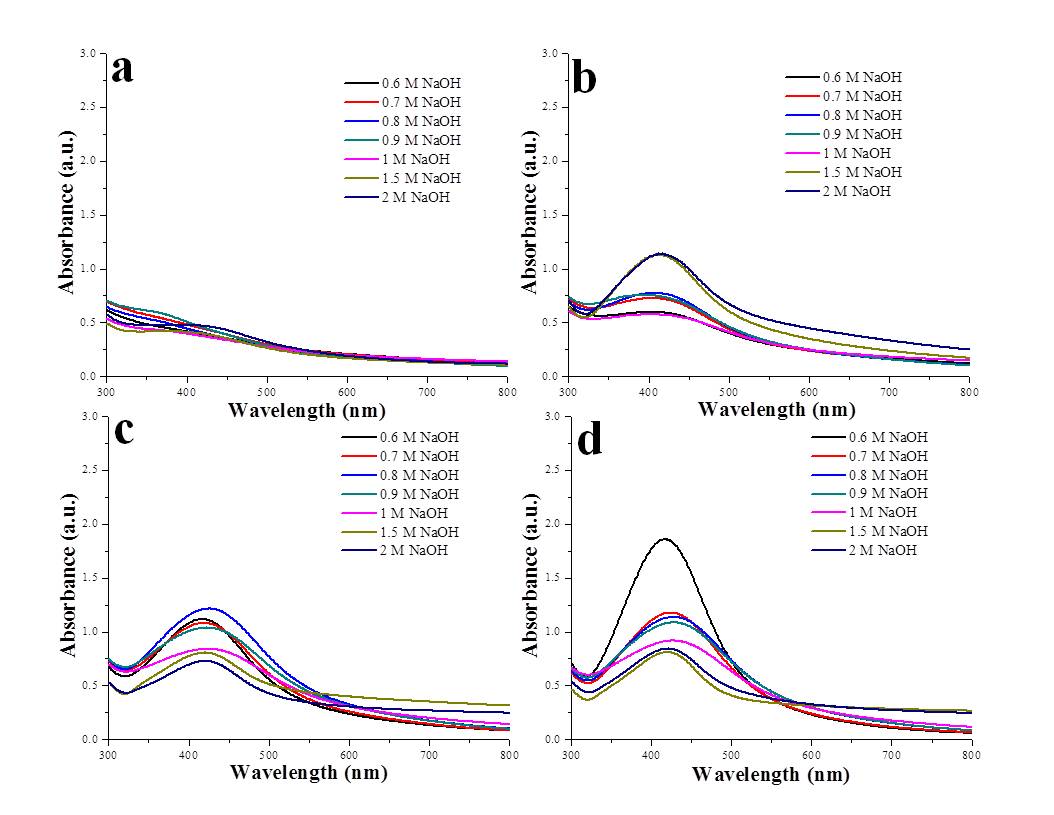


**Figure S1.1.** UV-visible absorption spectra of AL Ag NPs while optimizing the concentration of sodium hydroxide in day wise fashion; day 1(a), day 2 (b), day 3 (c), and day 4 (d).

**
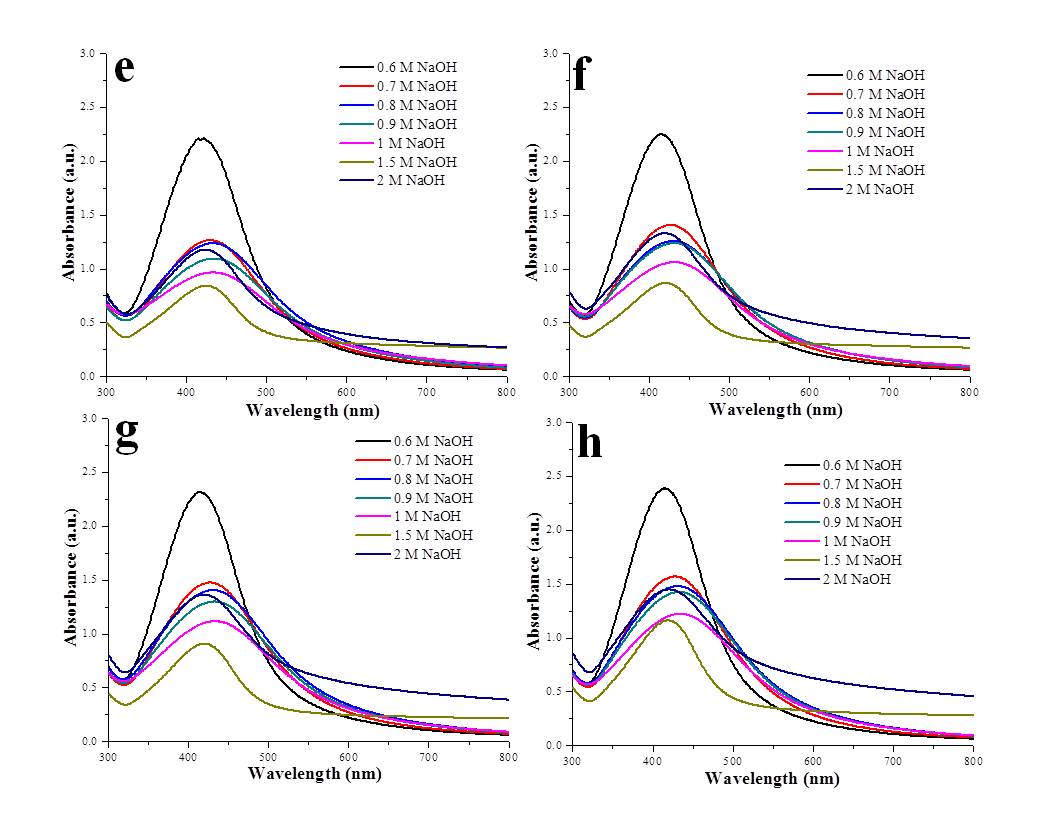
**

**Figure S1.2.** UV-visible absorption spectra of AL Ag NPs while optimizing the concentration of sodium hydroxide in day wise fashion; after (a), (b), (c), and (d); day 5(e), day 6 (f), day 7 (g), and day 8 (h).


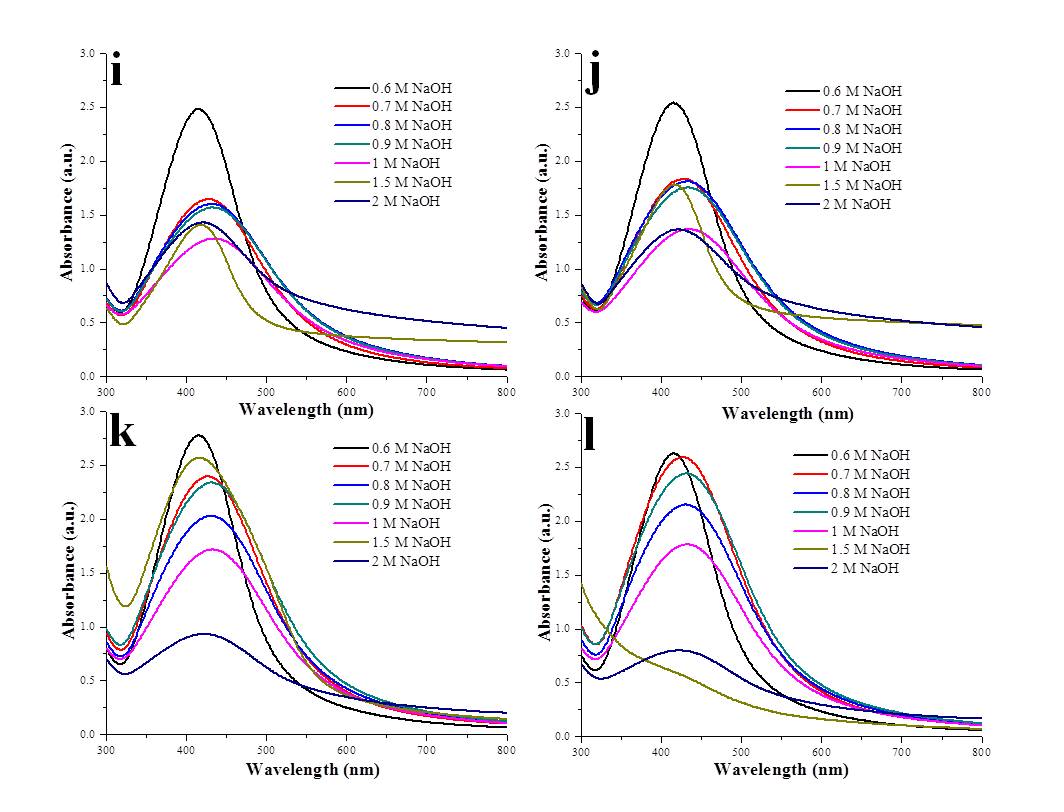


**Figure S1.3.** UV-visible absorption spectra of AL Ag NPs while optimizing the concentration of sodium hydroxide in day wise fashion; after (a), (b), (c), (d), (e), (f), (g), and (h); day 9(i), day 10 (j), day 14 (k), and day 15 (l).


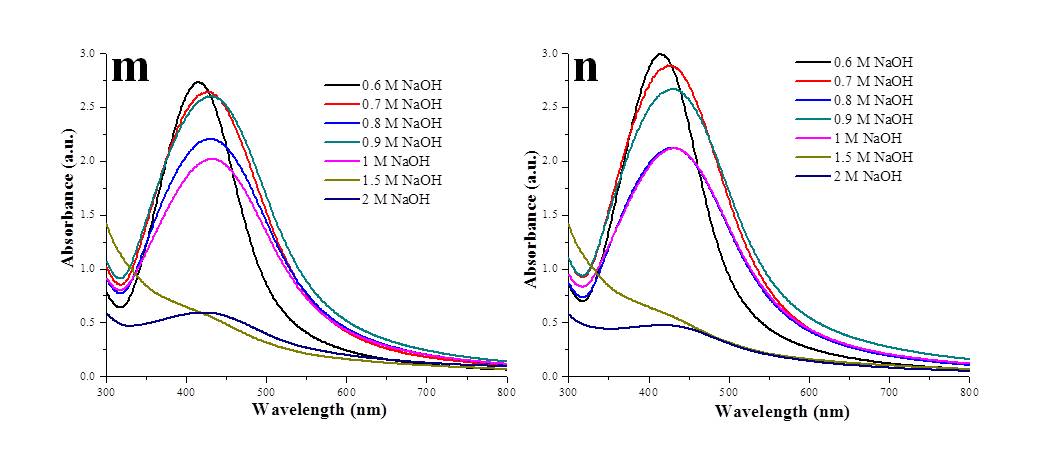


**Figure S1.4.** UV-visible absorption spectra of AL Ag NPs while optimizing the concentration of sodium hydroxide in day wise fashion; after (a), (b), (c), (d), (e), (f), (g), (h), (i), (j), (k), and (l); day 16(m), and day 17 (n).

**
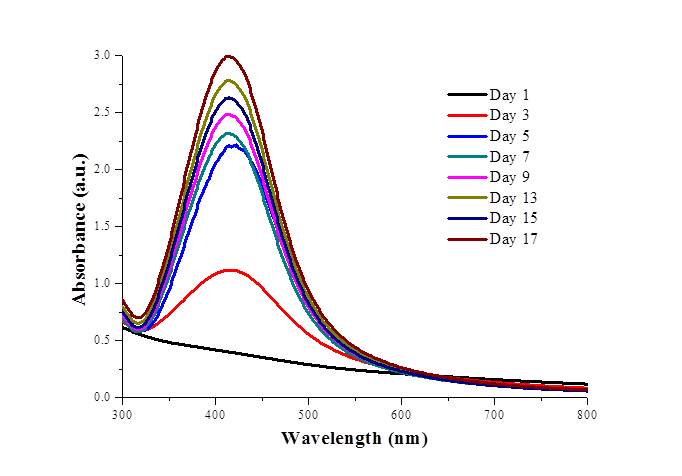
**

**Figure S2.** The evolution of absorption spectra under optimum conditions for AL Ag NPs.

.

**
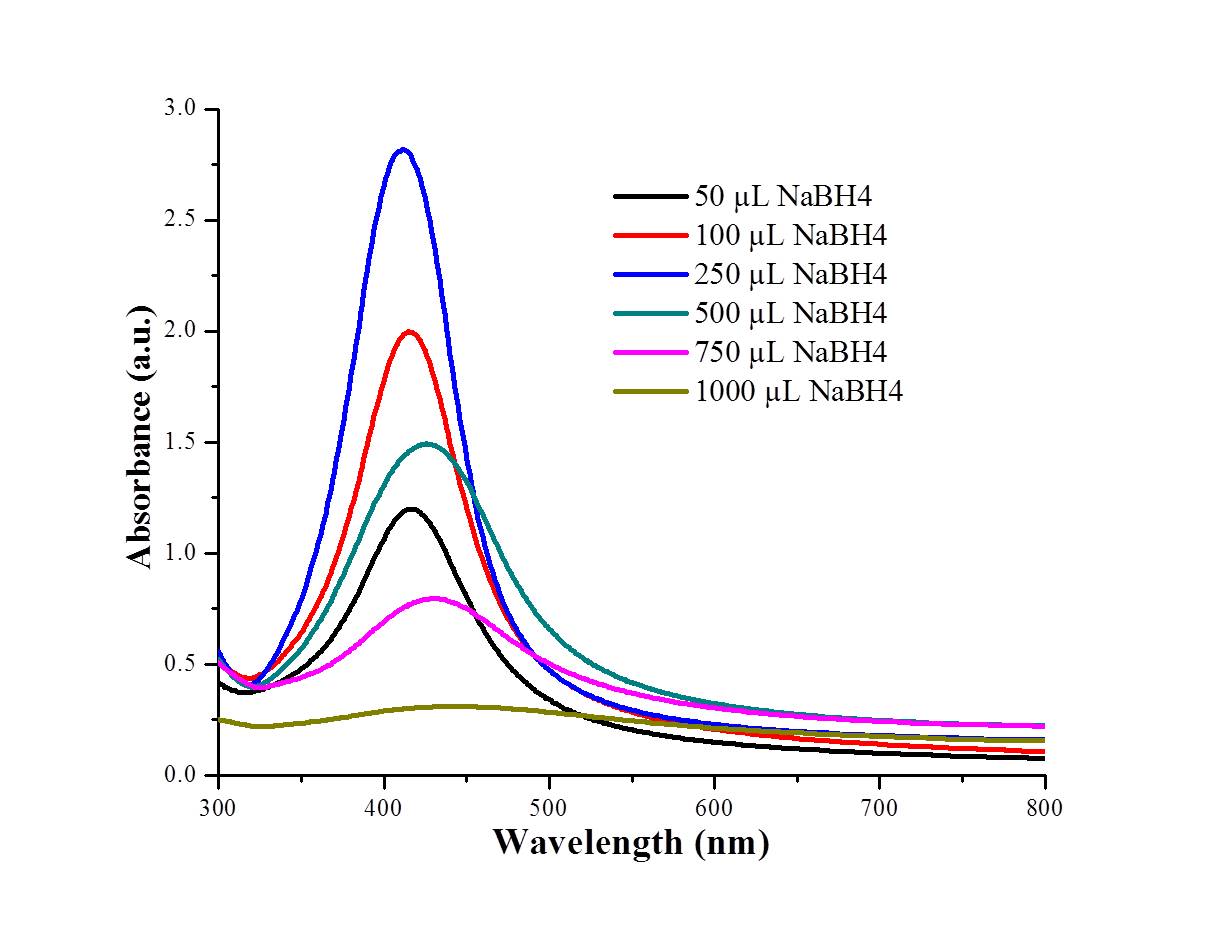
Figure S3.** UV-visible absorption spectra of BL Ag NPs while optimizing the concentration of sodium borohydride.

**
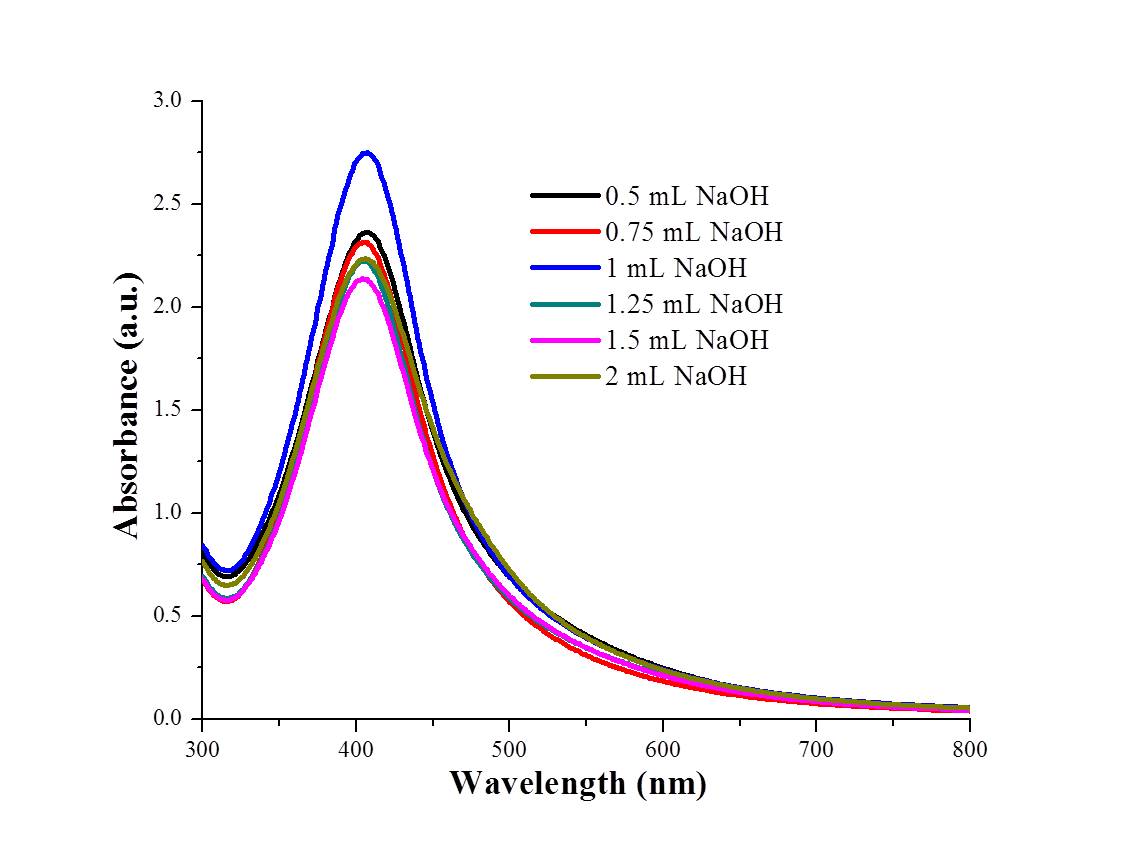
Figure S4.** UV-visible absorption spectra of RL Ag NPs while optimizing the concentration of sodium hydroxide.


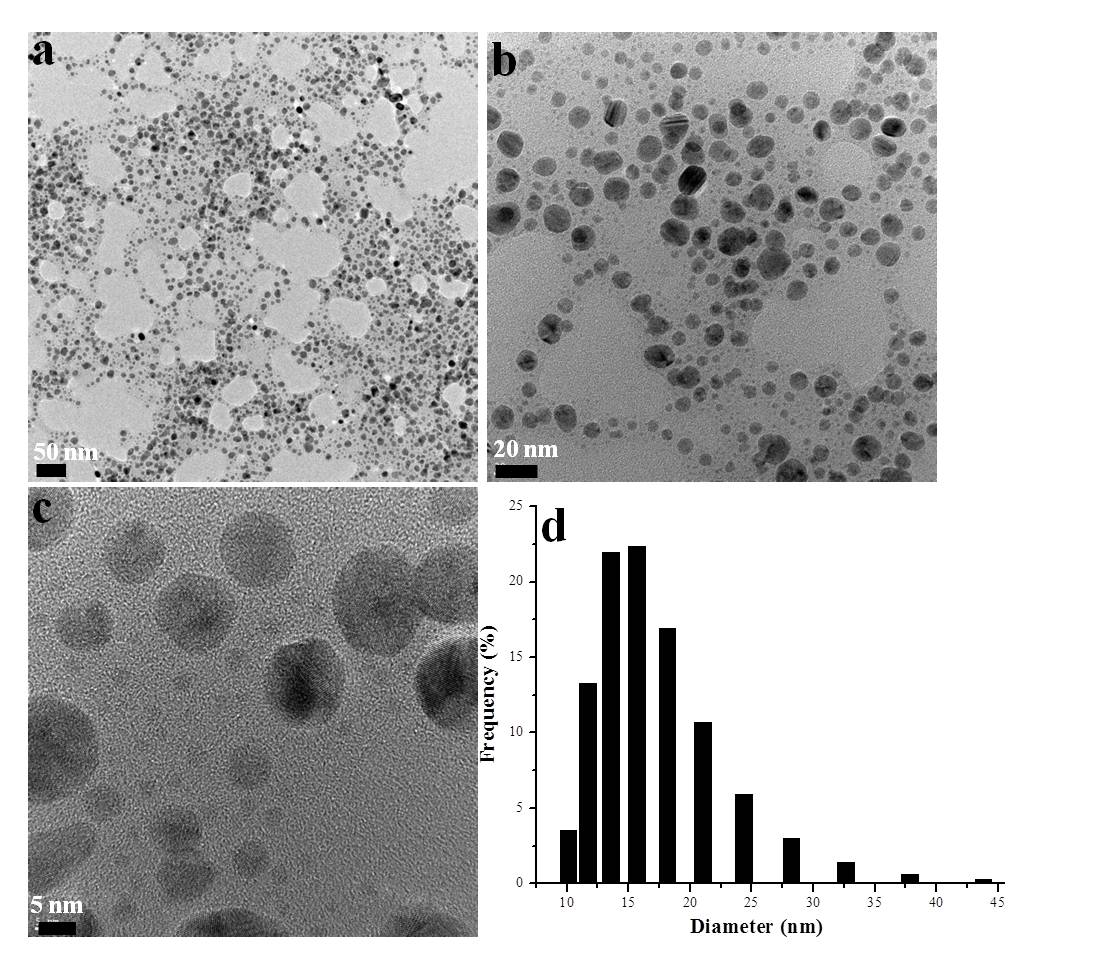


**Figure S5.** Transmission electron micrographs of AL Ag NPs at different magnifications. Scale bar corresponds to 50 nm (a), 20 nm (b), and 5 nm (c). Their size distribution determined by DLS is given in (d).

**Figure S6.** Transmission electron micrographs of BL Ag NPs at different magnifications. Scale bar corresponds to 50 nm (a), 20 nm (b), and 5 nm (c). Their size distribution determined by DLS is given in (d).


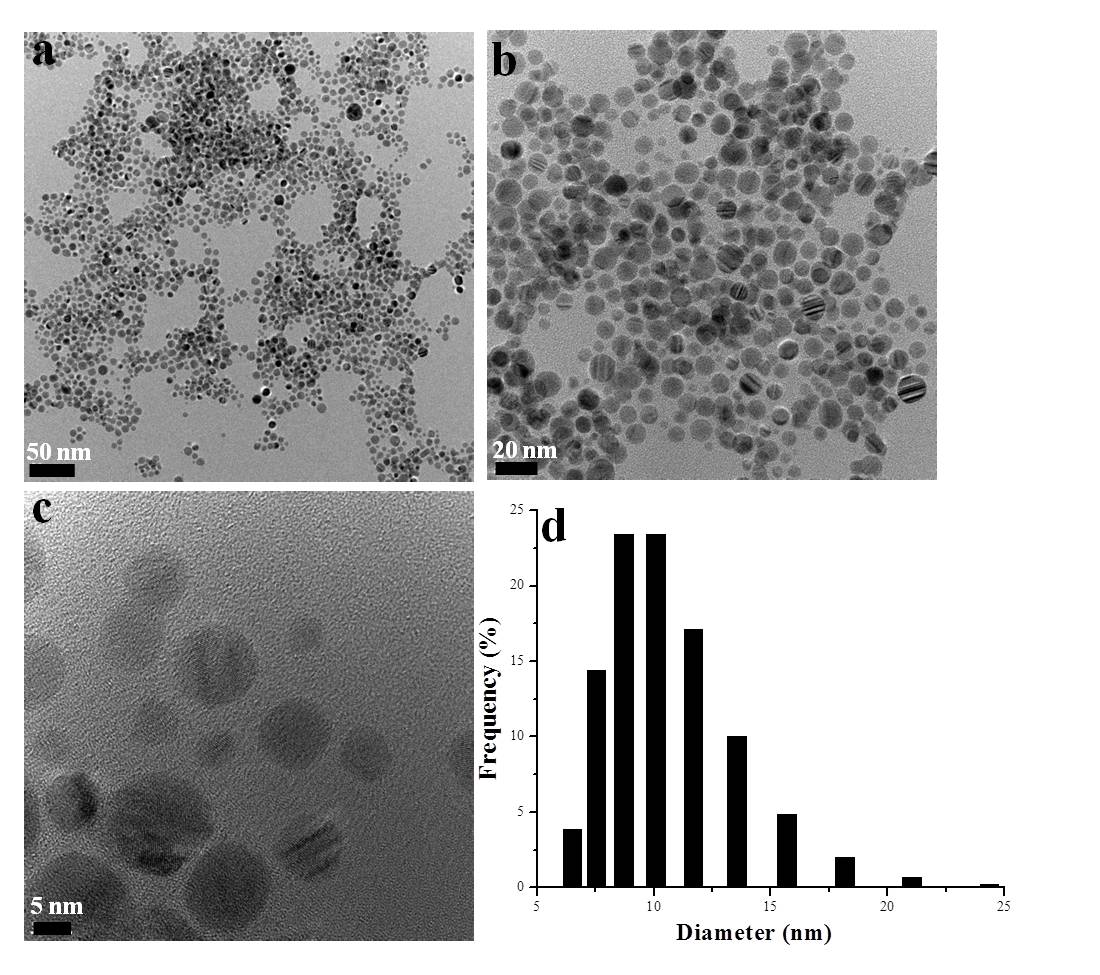


**Figure S7.** Transmission electron micrographs of RL Ag NPs at different magnifications. Scale bar corresponds to 50 nm (a), 10 nm (b), and 5 nm (c). Their size distribution determined by DLS is given in (d).


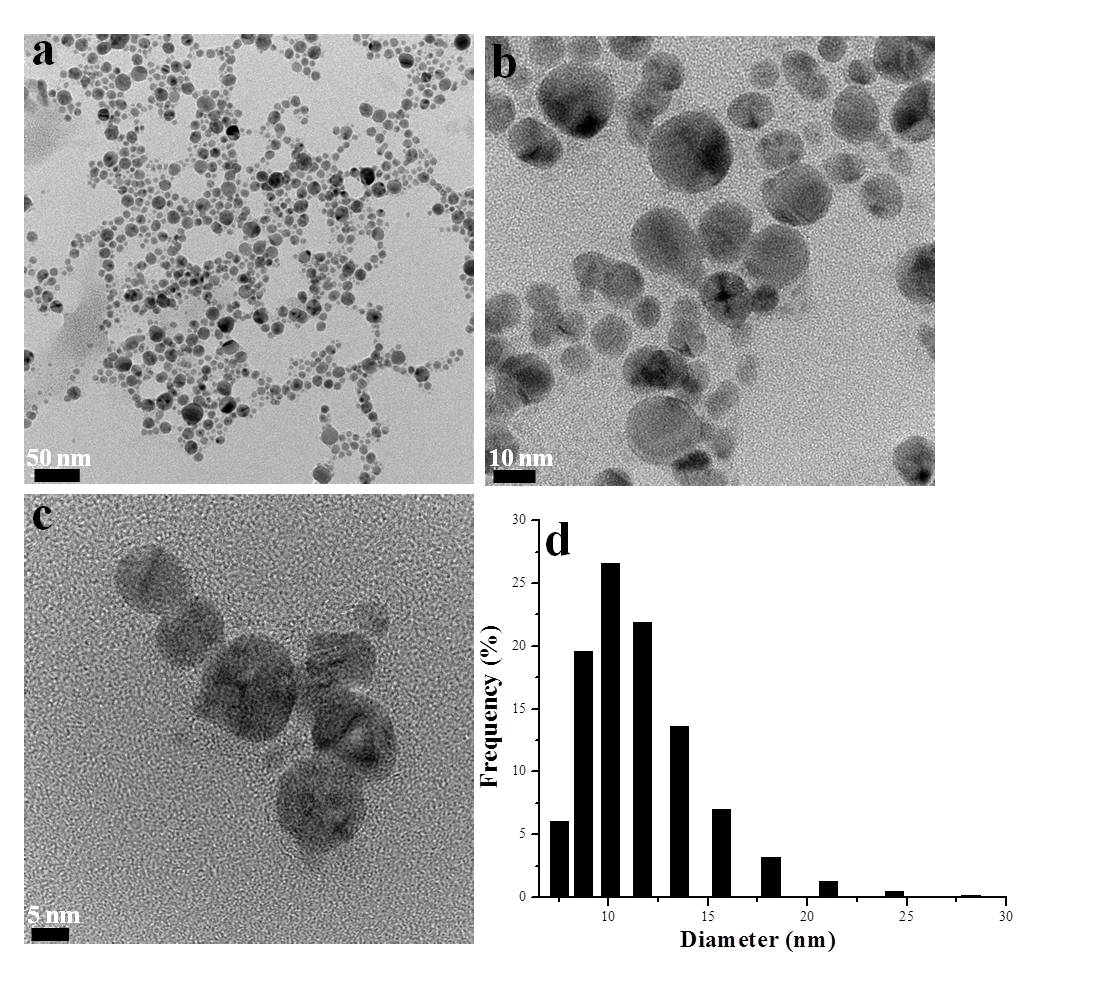


**
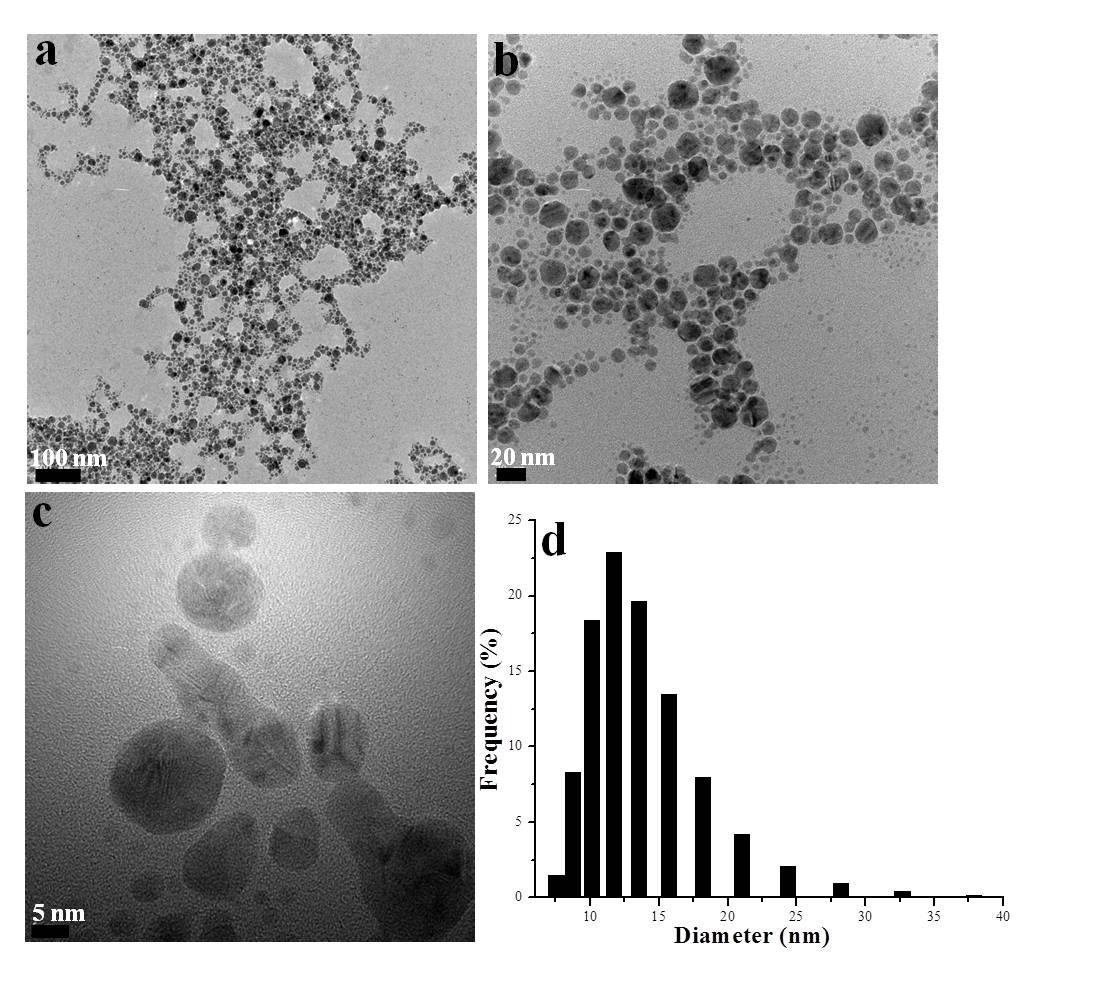
Figure S8.** Transmission electron micrographs of ML Ag NPs at different magnifications. Scale bar corresponds to 50 nm (a), 20 nm (b), and 5 nm (c). Their size distribution determined by DLS is given in (d).

**Table S1** Bactericidal effect of lysozyme stabilized Ag NPs against *P. aeruginosa 3*

(CFU in numbers, concentrations of particles applied in ppm)

CFU of *P. aeruginosa 3* (negative control) was calculated to be 1.758*10^17^.

| **Type of lysozyme stabilized Ag NPs** | **Concentration of particles**  **(ppm)** | **CFU in the presence of lysozyme stabilized Ag NPs** | **CFU in the presence of lysozyme**  **(positive control)** |
| --- | --- | --- | --- |
| AL Ag NPs | 100  80  60  40  20  10  1 | 0 ± 0.17  0 ± 0.21  2.20*10^2^ ± 0.18  2.60*10^2^ ± 0.24  2.5* 10^8^ ± 0.20  8.82*10^16^ ± 0.23  1.033*10^17^ ± 0.18 | 4.92*10^16^ ± 0.17  5.32*10^16^ ± 0.021  5.73*10^16^ ± 0.18  6.2* 10^16^ ± 0.24  8.1* 10^16^ ± 0.20  9.38*10^16^ ± 0.23  1.344*10^17^ ± 0.18 |
| BL Ag NPs | 100  80  60  40  20  10  1 | 0 ± 0.34  0 ± 0.29  3.6* 10^3^ ± 0.26  5.2* 10^5^ ± 0.30  4.92*10^12^ ± 0.33  1.086*10^17^ ± 0.29  1.272*10^17^ ± 0.31 | 6.11*10^16^ ± 0.34  6.69*10^16^ ± 0.29  7.54*10^16^ ± 0.26  8.78*10^16^ ± 0.30  9.8* 10^16^ ± 0.33  1.298*10^17^ ± 0.29  1.616*10^17^ ± 0.31 |
| RL Ag NPs | 100  80  60  40  20  10  1 | 0 ± 0.25  0 ± 0.24  0 ± 0.22  0 ± 0.26  7.3*10^5^ ± 0.27  1.079*10^17^ ± 0.23  1.346*10^17^ ± 0.24 | 7.32*10^16^ ± 0.25  7.84*10^16^ ± 0.24  8.215*10^16^ ± 0.22  9.0*10^16^ ± 0.26  9.98*10^16^ ± 0.27  1.195*10^17^ ± 0.23  1.547*10^17^ ± 0.24 |
| ML Ag NPs | 100  80  60  40  20  10  1 | 0 ± 0.19  0 ± 0.18  0 ± 0.19  3.4*10 ± 0.17  8.9*10^5^ ± 0.16  8.66*10^16^ ± 0.17  9.17*10^16^ ± 0.17 | 1.92*10^16^ ± 0.19  2.4*10^16^ ± 0.18  4.1*10^16^ ± 0.19  5.9*10^16^ ± 0.17  8.52*10^16^ ± 0.16  1.019*10^17^ ± 0.17  1.131*10^17^ ± 0.17 |

**Supplementary videos**

**Control_*P. aeruginosa 3*:** Video showing the growth of *Pseudomonas aeruginosa 3* in the absence of silver nanoparticles and lysozyme.

**AL_lysozyme control:** Video showing the growth of *Pseudomonas aeruginosa 3* in the presence of lysozyme (after one hour of incubation) used as control for AL Ag NPs.

**AL Ag NPs:** Video showing the growth of *Pseudomonas aeruginosa 3* in the presence of AL Ag NPs (after one hour of incubation).

**BL_lysozyme control:** Video showing the growth of *Pseudomonas aeruginosa 3* in the presence of lysozyme (after one hour of incubation) used as control for BL Ag NPs.

**BL Ag NPs:** Video showing the growth of *Pseudomonas aeruginosa 3* in the presence of BL Ag NPs (after one hour of incubation).

**RL_lysozyme control:** Video showing the growth of *Pseudomonas aeruginosa 3* in the presence of lysozyme (after one hour of incubation) used as control for RL Ag NPs.

**RL Ag NPs:** Video showing the growth of *Pseudomonas aeruginosa 3* in the presence of RL Ag NPs (after one hour of incubation).

**ML_lysozyme control:** Video showing the growth of *Pseudomonas aeruginosa 3* in the presence of lysozyme (after one hour of incubation) used as control for ML Ag NPs.

**ML Ag NPs:** Video showing the growth of *Pseudomonas aeruginosa 3* in the presence of ML Ag NPs (after one hour of incubation).
